# Blocking cholesterol formation and turnover improves cellular and mitochondria function in murine heart microvascular endothelial cells and cardiomyocytes

**DOI:** 10.3389/fphys.2023.1216267

**Published:** 2023-09-07

**Authors:** Alicja Braczko, Gabriela Harasim, Ada Kawecka, Iga Walczak, Małgorzata Kapusta, Magdalena Narajczyk, Klaudia Stawarska, Ryszard T. Smoleński, Barbara Kutryb-Zając

**Affiliations:** ^1^ Department of Biochemistry, Medical University of Gdansk, Gdańsk, Poland; ^2^ Laboratory of Electron Microscopy, University of Gdansk, Gdańsk, Poland

**Keywords:** statins, proprotein convertase subtilisin/kexin type 9 (PCSK9) inhibitor, endothelial cells, cardiomyocytes, mitochondria

## Abstract

**Background:** Statins and proprotein convertase subtilisin/kexin type 9 inhibitors (PCSK9i) are cornerstones of therapy to prevent cardiovascular disease, acting by lowering lipid concentrations and only partially identified pleiotropic effects. This study aimed to analyze impacts of atorvastatin and synthetic peptide PCSK9i on bioenergetics and function of microvascular endothelial cells and cardiomyocytes.

**Methods:** Mitochondrial function and abundance as well as intracellular nucleotides, membrane potential, cytoskeleton structure, and cell proliferation rate were evaluated in mouse heart microvascular endothelial cells (H5V) and cardiomyocytes (HL-1) under normal and hypoxia-mimicking conditions (CoCl_2_ exposure).

**Results:** In normal conditions PCSK9i, unlike atorvastatin, enhanced mitochondrial respiratory parameters, increased nucleotide levels, prevented actin cytoskeleton disturbances and stimulated endothelial cell proliferation. Under hypoxia-mimicking conditions both atorvastatin and PCSK9i improved the mitochondrial respiration and membrane potential in both cell types.

**Conclusion:** This study demonstrated that both treatments benefited the endothelial cell and cardiomyocyte bioenergetics, but the effects of PCSK9i were superior.

## 1 Introduction

Dyslipidemia is characterized by the increased levels of plasma total and low-density lipoprotein cholesterol (LDL-c) that may be accompanied by the reduced level of high-density lipoprotein cholesterol (HDL-c) (Berberich and Hegele, 2022). It has become a key target for the prevention and therapy of cardiovascular diseases. Among lipid-lowering therapies statins and more recently proprotein convertase subtilisin/kexin type 9 inhibitors (PCSK9i) are the most efficient drugs (Ott and Schmieder, 2009; Latimer et al., 2016).

Statins reduce a liver cholesterol production by the mechanism based on the inhibition of (3-hydroxy-3-methylglutaryl-coenzyme A) HMG-CoA reductase, the rate-limiting enzyme in cholesterol synthesis. Decreased concentration of cholesterol in hepatocytes enhances the expression of hepatic LDL receptors, which removes LDL and LDL precursors from the circulation (Maron et al., 2000). Apart from cholesterol-lowering effects, statins also have a wide range of well-documented pleiotropic effects including the improvement of the endothelial function, anti-inflammatory, anti-thrombotic, and immunomodulatory effects (Greenwood and Mason, 2007). The improvement of the endothelial function is predominantly related to beneficial effects of statins on nitric oxide (NO)/reactive oxygen species (ROS) balance and upregulation of endothelial nitric oxide synthase (eNOS), while anti-inflammatory effects are attributed to the regulation of cytokine production by statins (Bates et al., 2002; Tousoulis et al., 2007). Despite these benefits, long-term statin therapy is not optimal due to adverse reactions such as myopathy (Mach et al., 2018). Several lines of evidences have shown that mitochondrial dysfunction plays a role in statin-associated muscle symptoms (Mollazadeh et al., 2021). In skeletal muscle cells, statins may interfere with mitochondrial activity both via direct impairment of electron transport chain complexes, or via indirectly, as a consequence of mevalonate pathway metabolite depletion that includes isoprenoids and Coenzyme Q10 (CoQ10) (Muraki et al., 2012). However, despite side effects of statins in skeletal muscle mitochondria, the opposite trends were shown in cardiac mitochondria, where atorvastatin induced mitochondrial biogenesis and improved antioxidant capacity (Bouitbir et al., 2012). In myocardial ischaemia-reperfusion injury, statins reduced the severity of cardiomyocyte damage via the activation of mitochondrial ATP-sensitive potassium channels, uncoupling proteins, and inhibition of glycogen synthase kinase-3β phosphorylation (Bland et al., 2022). On the other hand, very limited data are available on the effects of statins in endothelial cell mitochondria. Conflicting reports have shown that atorvastatin improved their function in human primary umbilical vein endothelial cells (HUVEC), while decreasing CoQ10 content and enhancing mitochondrial ROS formation in human umbilical vein endothelial cell line (EA.hy926). (Ota et al., 2010; Broniarek et al., 2020).

PCSK9i disrupt the interaction between PCSK9 protein and membrane LDL receptor increasing the uptake of LDL by the cells (Roth and Davidson, 2018). There are several strategies that target the inhibition of PCSK9 such as monoclonal antibodies, synthetic small interfering RNA (siRNA) targetting PCSK9, vaccination, and small molecules (Nishikido and Ray, 2018). It was shown that similar to statins, PCSK9i demonstrates pleiotropic effects beyond its lipid-lowering action. Several reports demonstrated that PCSK9i dampens the pro-inflammatory activation of vascular cells, attenuates plaque oxidative stress, decreases proliferation and migration of vascular smooth muscle cells, reduces endothelial cell apoptosis, and limits thrombosis, ultimately reducing the risk of a clinical event related to atherosclerotic plaque (Li et al., 2017; Punch et al., 2022; Wang et al., 2023). However, still little is known about their effects on the mitochondria, especially in vascular and cardiac cells.

Murine *in vivo* or cellular models are useful for studying atherosclerosis ethology, pathogenesis, and complications, providing a valuable platform for testing different pharmacological approaches (Lee et al., 2017). Recently, we have documented an impairment in cardiac mitochondria function in mouse model of human-like dyslipidemia (Braczko et al., 2022). The aim of this work was to investigate the pleiotropic effects of statins and PCSK9i on mitochondrial and cellular function of cardiac endothelial cells and cardiomyocytes.

## 2 Materials and methods

### 2.1 Cell culture and treatment

Murine heart microvascular endothelial cells (H5V) were kindly provided by Dr. Patrycja Koszałka (Department of Cell Biology, Faculty of Medical Biotechnology, Medical University of Gdańsk, Gdańsk, Poland). H5V cells were cultured in DMEM supplemented with 4.5 g/L glucose, stable glutamine, sodium pyruvate and sodium bicarbonate (cat.no. D0822, Merck, Germany), 10% (v/v) fetal bovine serum (FBS) (cat.no. 10500064, Gibco, Brazil), and 1% (v/v) penicillin/streptomycin (cat.no. 30-002-CI, Corning, Manassas, VA, United States of America). Murine cardiac muscle cell line HL-1 (cat. No. SCC065) was purchased from Merck, Germany, and cultured in gelatin/fibronectin coated flasks (fibronectin-F1141, Merck, Germany; EmbryoMax 0.1% Gelatin Solution cat. no. ES-006, Merck, Germany) in Claycomb Medium (cat.no. 51800C, Merck, Germany) supplemented with 0.1 mM norepinephrine (cat.no. A0937, Merck, Germany), 0.3 mM L-Ascorbic Acid (cat.no. A7506, Merck, Germany), 10% (v/v) FBS (cat.no. 10500064, Gibco, Brazil), 2 mM L-glutamine (cat.no. 25-005-CV, Corning, Manassas, VA, United States) and 1% (v/v) penicillin/streptomycin (cat.no. 30-002-CI, Corning, Manassas, VA, United States). Both cell lines were cultured in a completely humid atmosphere at 37°C and 5% CO2.

Determination of intracellular nucleotides and structure of mitochondria in H5V and HL-1 cells were performed at 24-well plates on subconfluent monolayer (80%) after the third passage. In order to assess cell proliferation, HL-1 and H5V cells were seeded on 24-well-plates on sparse monolayer about 30%. The cells were pre-treated for 30 min with 0.1, 0.3, and 0.5 mM atorvastatin (cat.no. 134523-03-8, Cayman Chemicals, United States) or 100, 300, and 500 nM PCSK9i, a synthetic LDL receptor epidermal growth factor-like repeat A (EGF-A) domain peptide (cat.no. 5087610001, Merck, Germany). Dimethyl sulfoxide (DMSO, cat. no. D2438, Merck, Germany) was used as vehicle for atorvastatin, and was added at a final concentration of 0.5% (v/v) to all remining treatments. Then, to mimic hypoxia conditions, cells were treated with 100 μM cobalt chloride (CoCl_2_, cat. no. C8661, Merck, Germany) for 24 h at 37°C and 5% CO_2_. PCSK9i and CoCl_2_ were reconstituted in sterile water, which was used in control and atorvastatin-treated cells at the corresponding volumes. To induce hypoxic conditions, HL-1 cells were placed in hypoxic chamber for 24 h. The concentration of the oxygen in atmosphere of hypoxic chamber was 1%.

### 2.2 Intracellular nucleotides concentration

After the atorvastatin and PCSK9i treatment with and without CoCl_2_ on 24 well plates, H5V and HL-1 cell monolayers were washed with HBSS (cat. No. 21-023-CV, Corning, Manassas, VA, United States) and ice-cold 0.4 M HClO4 (300 μL) was added. Next, the plate was frozen at −80°C. 24 h later, the plate was thawed on ice and frozen again. After next thawing on ice, supernatants were collected and neutralized to pH 6 by adding 3 M K3PO4. Subsequently, samples were centrifuged (20,800 × *g*, 4°C, 10 min) and the supernatants were collected and analysed for nucleotide concentrations with UHPLC (Kutryb-Zajac et al., 2022b). The concentrations of nucleotides were expressed as nmol/mg of protein.

### 2.3 Mitochondrial function

The analysis of mitochondria function was performed in H5V and HL-1 cells using a Seahorse XFp analyzer (Agilent, Santa Clara, CA, United States). Cells were seeded in XFp 8-well microplates (5 × 104 cells/well) in a final volume of 200 μL DMEM full medium (cat. No. D0822, Merck, Germany). The next day, cells were pre-treated with 0.1 mM atorvastatin and 500 nM PCSK9i. Control cells were treated with vehicle (0.5% v/v DMSO). After 30 min pre-incubation, cells were incubated with 100 μM CoCl2 or sterile water for a further 24 h. Then, cells were washed and incubated in 180 μL of Seahorse DMEM medium (enriched with 1 mM pyruvate, 2 mM glutamine, and 10 mM glucose) at 37°C for 45 min. Then, the analyser sequentially injected oligomycin (inhibitor of Complex V), carbonyl cyanide-p-trifluoromethoxyphenylhydrazone (FCCP; the mitochondrial uncoupler), and a mix of antimycin A (inhibitor of Complex III) and rotenone (inhibitor of Complex I) to a final concentration of 1.5 μM, 1 μM, and 0.5 μM, respectively. Subsequently, the oxygen consumption rate (OCR) was recorded. ATP-linked, basal, maximal and non-mitochondrial respiration, and proton leak were calculated.

### 2.4 Cell proliferation test

Proliferation rate assay was performed on H5V and HL-1 cells seeded at 0.02 × 106 on 96-well cell imaging plate (cat. No. CC315, Merck, Germany), pre-treated with 0.1 mM atorvastatin, 500 nM PCSK9 inhibitor, or vehicle (0.5% v/v DMSO), following 24 h incubation with or without CoCl2. The assessment of proliferation rate was performed for using Axio Observer 7 inverted microscope with differential interference contrast and ZEN software (Carl Zeiss Inc., Dresden, Germany). Images were generated every 1 h and analysed using ImageJ2 software (Fiji package). (Schindelin et al., 2012).

### 2.5 Immunofluorescence staining

Immunofluorescent staining of endothelial total and phosphorylated at Ser1177 nitric oxide synthase (total-eNOS and phospho-eNOS), nitric oxide, mitochondrial TOM20 protein, and actin filaments was performed on H5V cells plated in 96-well format (cat. No. CC315, Merck, Germany) at 0.01 × 106 cells/well. Cells were fixed in 4% formaldehyde in phosphate buffered saline (PBS, cat. No. P2272, Merck, Germany) for 15 min at RT, rinsed with PBS, permeabilized with 0.1% Triton X-100 (cat. No. T8787, Merck, Germany) for 10 min, rinsed with PBS, and incubated for 30 min in blocking PAD solution, which included bovine serum albumin (BSA, 1% v/v, cat. No. A1595, Merck, Germany) and normal goat serum solution in PBS (10% v/v, cat. No. G9023, Merck, Germany). Cells were incubated with rabbit primary polyclonal anti-eNOS or phospho-eNOS-Ser1177 monoclonal antibody (cat. No. PA031A/MA5-14957, Thermo Fisher Scientific, Waltham, MO, United States, 1:250) for 1 h. After washing, Alexa Fluor 594- or 488-conjugated goat-anti-rabbit secondary antibody (cat. No. A-11012/A-11008, Jackson Immuno, Cambridgeshire, United Kingdom, 1:600) was added for 30 min. DAF-FM Diacetate (cat. No. D23842, Thermo Fisher Scientific, Hertfordshire, United Kingdom) was used to detect NO in living cells. Cells were incubated with DAF-FM diacetate at 10 μM final concentration for 30 min. Alternatively, H5V cells were incubated with mouse monoclonal primary anti-TOM20 antibody (cat.no. sc-17764, Santa Cruz Biotechnology, Dallas, TX, United States, 1:50) for 1 h, followed by 30 min incubation with Alexa Fluor 594-conjugated goat-anti-mouse secondary antibody (cat.no. A-11005, Jackson Immuno, Cambridgeshire, United Kingdom, 1:600) and 45 min incubation with Phalloidin-Atto 488 conjugate (cat. No. 49409), Merck, Germany). In order to assess mitochondrial function, after the treatment, living cells were stained with MitoTracker Deep Red FM (cat. No. M22426, Thermo Fisher Scientific, Hertfordshire, United Kingdom) at final concentration of 0.5 μM for 45 min, then washed twice with PBS and fixed in 4% formaldehyde in PBS as described earlier. Cell nuclei were counterstained by DAPI (cat. No. MBD0015, Merck, Germany) in all experiments.

On HL-1 cells, immunofluorescent staining of mitochondria, TOM20 protein and actin filaments was performed. Cells were plated in 96-well format (cat. No. CC315, Merck, Germany) at 0.015 × 106 cells/well and living cells were stained with 500 nM MitoTracker Deep Red FM as described above. Alternatively, cells were fixed and stained using primary anti-TOM20 antibody and Phalloidin-Atto 488 conjugate as described previously. Cell nuclei were counterstained by DAPI (MBD0015, Merck, Germany). Images were taken and analysed using Axio Observer 7 inverted fluorescence microscope (Carl Zeiss Inc., Dresden, Germany) and ZEN software v.3.3 blue edition (Carl Zeiss Inc., Dresden, Germany).

### 2.6 Electron microscopic analyses

Cells were fixed in ice-cold 2.5% electron microscopy grade glutaraldehyde (Agar) in 0.1 M PBS (pH 7.4) then post-fixed in 1% osmium tetroxide (Agar) for 1 h. Smaples were dehydrated through a graded series of ethanol (30%–100%), and embedded in Epon (Sigma). Ultrathin (65 nm) sections were cut using a Leica UC7 ultramicrotome were stained with Uranyless (Delta Microscopies) and Reynold’s lead citrate (Delta Microscopies), and examined on a Tecnai G2 Spirit BioTWIN transmission electron microscope at 120 kV.

### 2.7 Statistical analysis

Statistical analysis was performed using InStat software (GraphPad, San Diego, CA, United States). Primarily, to estimate the normality distribution, we accomplished the normality tests. The comparisons of mean values between groups were evaluated by unpaired Student’s t-test or two-way Anova followed by Holm-Sidak post-hoc test, as appropriate. Error bars showed the standard error of the mean (SEM). The exact value of n was provided for each type of experiments. The statistical significance was assumed at *p* ≤ 0.05.

## 3 Results

### 3.1 Effect of atorvastatin and PCSK9i on intracellular nucleotide concentration in endothelial H5V cells

**FIGURE 1:**
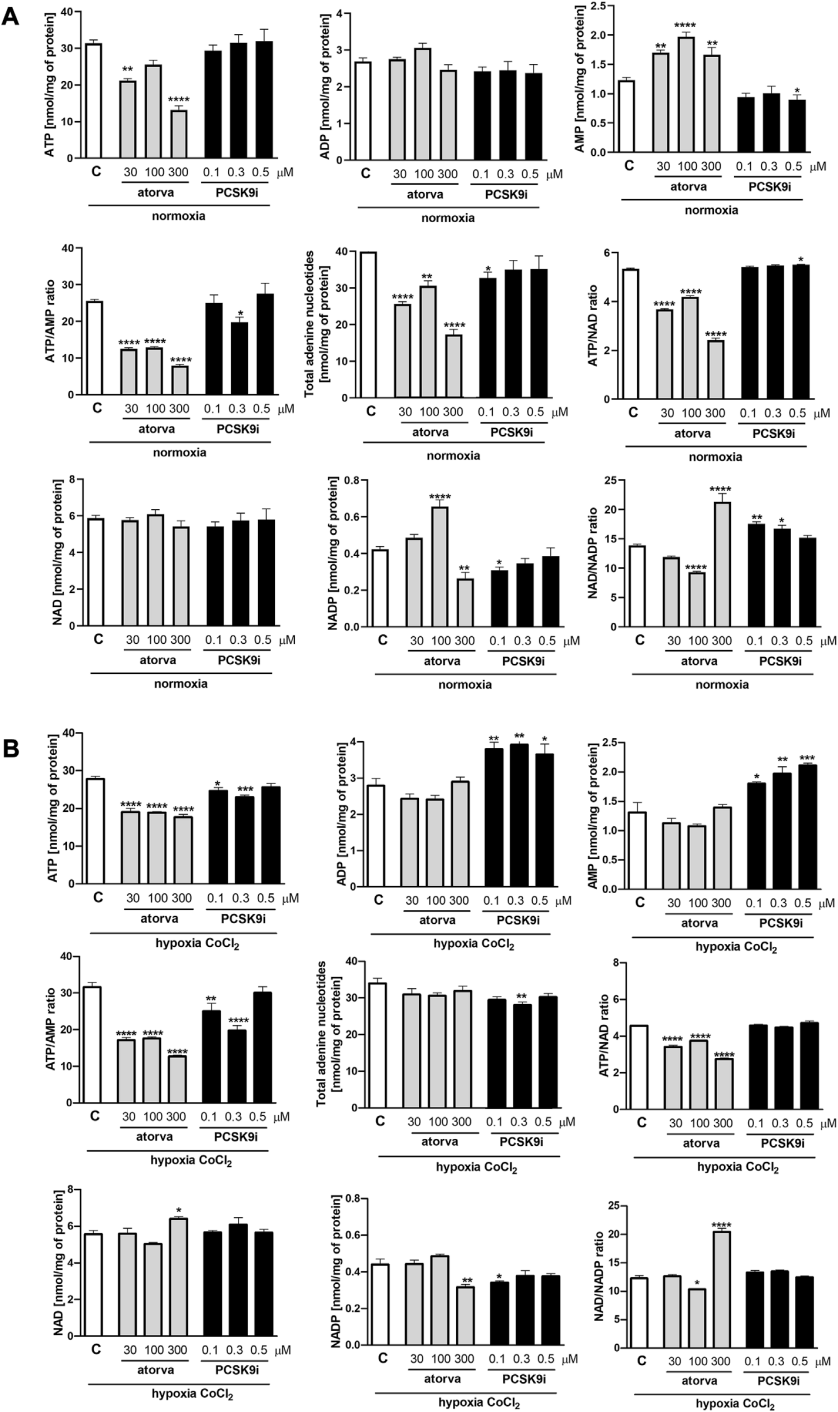
Intracellular nucleotide concentration in murine heart endothelial cells (H5V) after atorvastatin and PCSK9 inhibitor (PCSK9i) treatment in normoxia and hypoxia-mimicking conditions. The concentrations of adenosine triphosphate (ATP), adenosine diphosphate (ADP), adenosine monophosphate (AMP), nicotinamide adenine dinucleotide (NAD^+^), nicotinamide adenine dinucleotide phosphate (NADP) and the ratios of ATP/ADP and ATP/NAD + after 0.03, 0.1 and 0.3 mM atorvastatin and 100, 300, and 500 nM PCSK9i treatment under normoxia **(A)**, CoCl_2_-mimicking hypoxia (0.1 mM CoCl_2_, 24 h) **(B)** conditions. Values are shown as mean ± SEM; *n* = 6; *p < 0.05, **p < 0.01, ***p < 0.001, ****p < 0.0001 vs. untreated cells in normoxia.

First, we determined nucleotides concentration in murine endothelial cells (H5V) treated with atorvastatin (30, 100 and 300 µM) and PCSK9 inhibitor (0.1, 0.3 and 0.5 µM) under normoxia and hypoxia-mimicking conditions. Cell treatment with 30 μM and 300 µM atorvastatin for 24 h caused a decrease in ATP concentration in comparison to control cells (treated with DMSO) under normoxia (Figure 1A). Additionally, atorvastatin-treatment induced an increase in AMP concentration, diminished ATP/AMP ratio, total adenine nucleotides pool (TAN), and ATP/NAD ratio in H5V cells as compared to control under normoxia (Figure 1A). Conversely, 24 h 0.5 µM PCSK9i-treatment contributed to preserved ATP concentration, reduction in AMP level, unaltered ATP/AMP ratio and TAN, and increased in ATP/NAD ratio in comparison to control under normoxia (Figure 1A). Next, we repeated the experiment under hypoxia-mimicking conditions by treatment with 100 µM CoCl_2_ for 24 h (Figure 1B). Similar to normoxia, we revealed diminished ATP concentration, ATP/AMP and ATP/NAD ratio after atorvastatin treatment in H5V cells as compared to control (Figure 1B). Nevertheless, ADP, AMP and TAN level remained unchanged after atorvastatin treatment in hypoxia-mimicking condition (Figure 1B). Interestingly, H5V cells treated with PCSK9i exhibited preserved ATP, ADP, TAN and NAD concentration and ATP/AMP ratio as compared to control under normoxia conditions (Figure 1A). However, in hypoxia-mimicking conditions, endothelial cells after treatment with 0.5 µM PCSK9i were characterized by unaltered ATP, TAN and NAD concentration and ATP/AMP ratio relative to control (Figure 1B).

### 3.2 Effect of atorvastatin and PCSK9i on mitochondria in H5V cells

**FIGURE 2:**
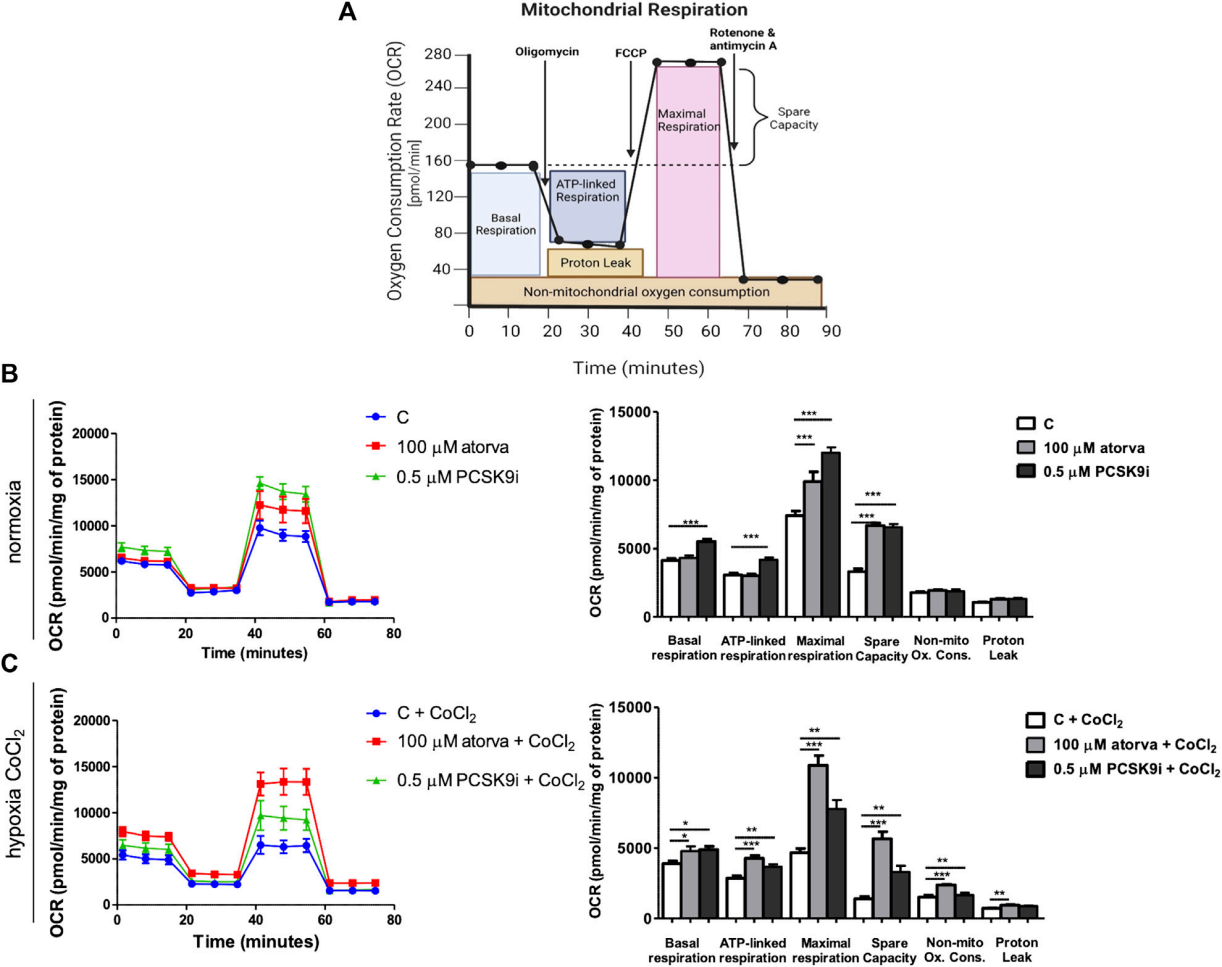
Mitochondria function in murine heart endothelial cells (H5V) after atorvastatin and PCSK9 inhibitor (PCSK9i) treatment under normoxia and hypoxia-mimicking conditions. Scheme of MitoStress Test created with BioRender.com **(A)**, Oxygen consumption rate (OCR) as presented by the Seahorse XF Extracellular Flux Analyzer after the sequential injection of oligomycin, carbonyl cyanide-p-trifluoromethoxyphenylhydrazone (FCCP) and mix of rotenone and antimycin A, and parameters of mitochondrial function in control (vehicle-pre-treated) cells and cells pre-treated with 100 µM atorvastatin or 0.5 µM PCSK9 inhibitor, after following incubation in normoxia **(B)** and CoCl_2_-mimicking hypoxia (24 h with 0.1 mM CoCl_2_) **(C)** conditions. Values are shown as mean ± SEM; *n* = 4; *p < 0.05, **p < 0.01, ***p < 0.001, ****p < 0.0001.

To elucidate the impact of statins and PCSK9i on bioenergetics of endothelial cells, we examined mitochondrial function in H5V cells after treatment with atorvastatin and PCSK9i (100 µM and 0.5 µM, respectively) in normoxia and hypoxia-mimicking conditions. We revealed an improvement of mitochondrial function in endothelial cells after treatment with atorvastatin and PCSK9i under normoxia and hypoxia-mimicking conditions (Figures 2B,C). H5V cells treated with the PCSK9 inhibitor exhibited an increase in parameters as basal respiration, ATP linked respiration, maximal respiration and spare capacity as compared to control under normoxia (Figure 2B). However, atorvastatin-treatment cells contributed only to an increase in maximal respiration and spare capacity in the same conditions (Figure 2B). Interestingly, under hypoxia-mimicking conditions, both atorvastatin- and PCSK9i-treatment improved maximal respiration, spare capacity, ATP-linked respiration, basal respiration and non-mitochondrial oxygen consumption in H5V cells relative to the control (Figure 2C).

**FIGURE 3:**
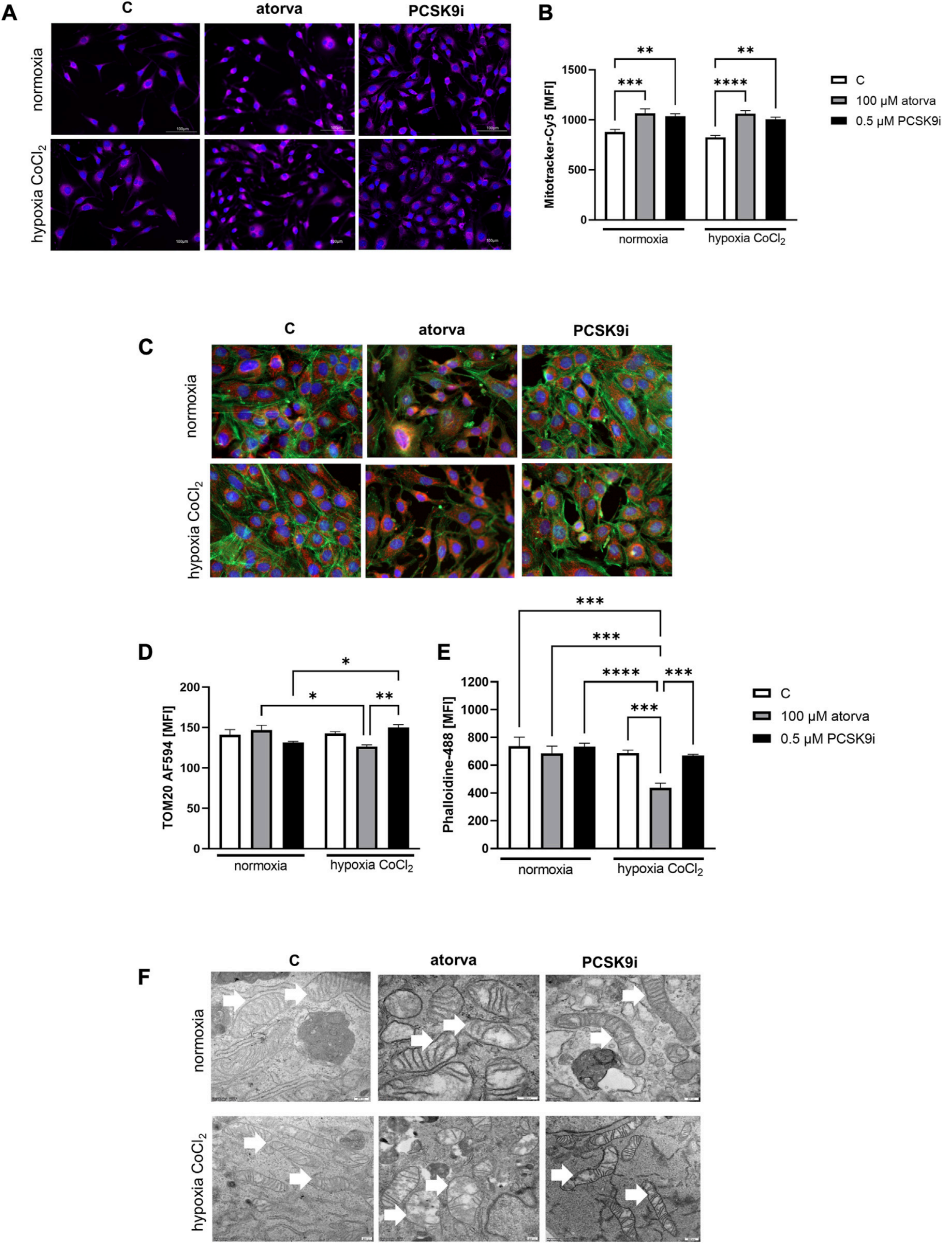
Mitochondria abundance and membrane potential in murine heart endothelial cells (H5V) after atorvastatin and PCSK9 inhibitor (PCSK9i) treatment under normoxia and hypoxia-mimicking conditions. Representative images of Mitotracker-Cy5 fluorescence staining **(A)** and quantitative analysis of mean fluorescence intensity (MFI) for Mitotracker-Cy5 in control (vehicle-pre-treated) cells and cells pre-treated with 100 µM atorvastatin or 0.5 µM PCSK9 inhibitor, after following incubation under normoxia and CoCl_2_-mimicking hypoxia (24 h with 0.1 mM CoCl_2_) conditions **(B)**. Representative images for TOM20-AF594 (red), α-actin-Phalloidin488 (green) and nuclei-DAPI (blue) staining analysed using fluorescence microscope **(C)** and quantitative analysis of MFI for TOM20-AF584 **(D)**. Quantitative analysis of mean fluorescence intensity for α-actin-Phalloidin488 **(E)**. Representative images of endothelial cell mitochondria in control cells and cells pre-treated with 100 µM atorvastatin or 0.5 µM PCSK9 inhibitor, after following incubation under normoxia and CoCl_2_-mimicking hypoxia (24 h with 0.1 mM CoCl_2_) conditions **(F)**. Values are shown as mean ± SEM; *n* = 6; *p < 0.05, **p < 0.01, ***p < 0.001, ****p < 0.0001.

Subsequently, we investigated mitochondrial membrane potential by Mitotracker-Cy5 fluorescence staining and determined mitochondrial abundance by TOM20 protein in H5V cells treated with atorvastatin and PCSK9i in normoxia and hypoxia-mimicking conditions. H5V cells treated with both, 100 µM atorvastatin and 0.5 µM PCSK9i, were characterized by increased mitochondrial membrane potential under hypoxia-mimicking conditions and normoxia as compared to control (Figures 3A, B). Next, by immunofluorescence staining, we determined TOM20 protein, a biomarker of mitochondrial abundance, in H5V cells treated with atorvastatin and PCSK9i under both conditions. Quantitative analysis of mean fluorescence intensity (MFI) revealed increased expression of TOM20 after cell treatment with PCSK9i as compared to atorvastatin-treated cells under hypoxia-mimicking conditions (Figures 3C, D).

Next, we investigated the ultrastructure of mitochondria in endothelial cells after treatment with PCSK9i and atorvastatin, by electron microscope under normoxia and hypoxia-mimicking conditions. Mitochondria in H5V cells were swollen and in irregular shape and cristae after treatment with atorvastatin. PCSK9i-cell treatment caused elongated mitochondrial structure in H5V cells under both conditions (Figure 3F).

### 3.3 Effects of atorvastatin and PCSK9i treatment on α-actin cytoskeleton and proliferation rate in H5V endothelial cells

**FIGURE 4:**
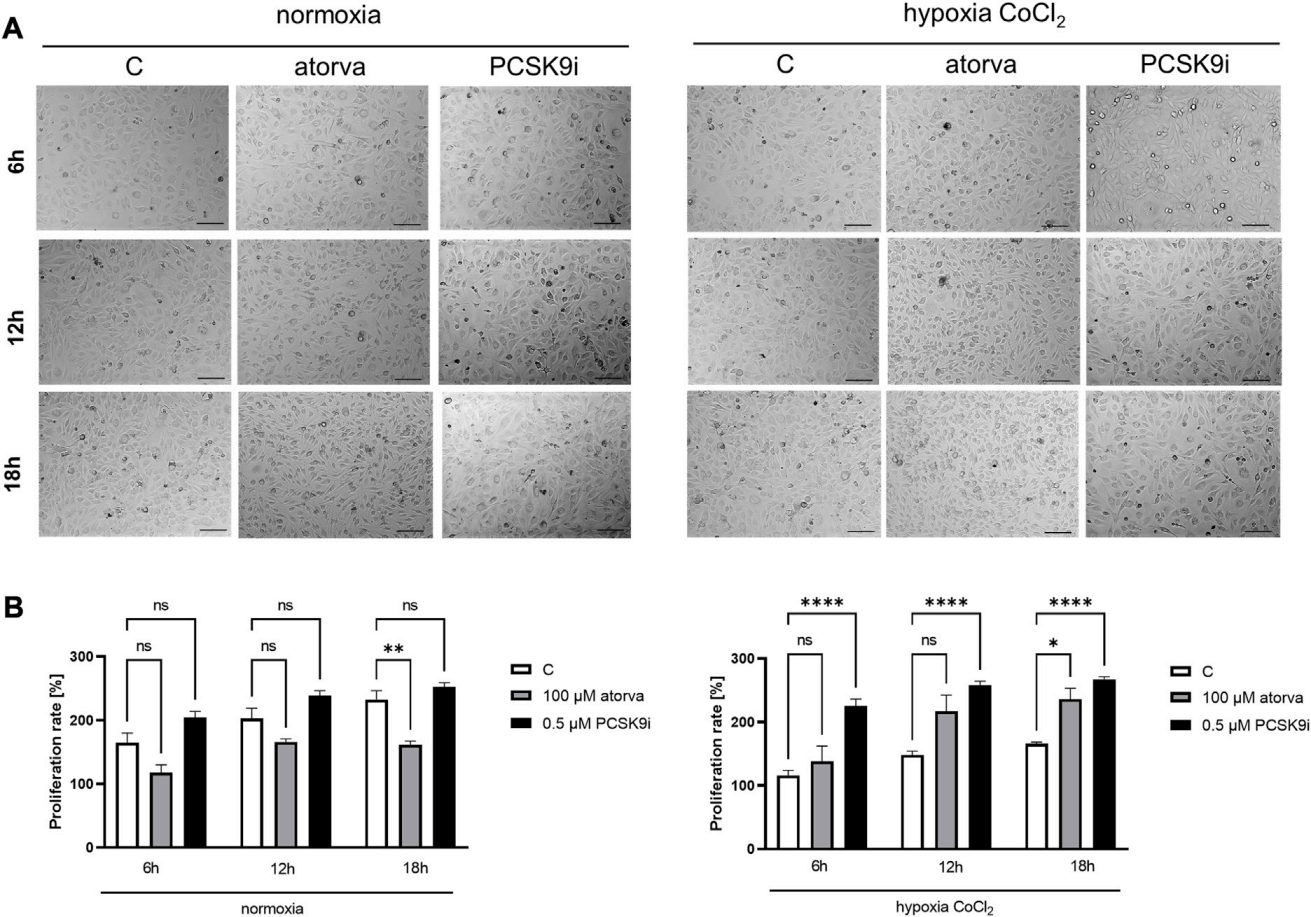
Proliferation rates of murine heart endothelial cells (H5V) in the presence of atorvastatin and PCSK9i under normoxia and hypoxia-mimicking conditions. Representative images **(A)** and quantitative results of the proliferation rates of control (vehicle-pre-treated) cells and cells pre-treated with 100 µM atorvastatin or 0.5 µM PCSK9 inhibitor, after following incubation in normoxia and CoCl_2_-mimicking hypoxia (24 h with 0.1 mM CoCl_2_) conditions **(B)**. Values are shown as mean ± SEM; *n* = 6; *p < 0.05, **p < 0.01, ****p < 0.0001.

To establish actin filaments in the cytoskeleton, phalloidin staining of H5V cells was performed after treatment with atorvastatin and PCSK9 inhibitor under normoxia and hypoxia-mimicking conditions. Analysis of phalloidin MFI disclosed cytoskeleton disorganization in H5V cells treated with atorvastatin in comparison to PCSK9i-treated cells and control in hypoxia-mimicking conditions (Figures 3C, E). Then, we evaluated the effects of drugs on endothelial cell proliferation after PCSK9i or atorvastatin treatment under normoxia and hypoxic conditions using a light microscope. We revealed that under normoxia, atorvastatin slowed the proliferation of H5V cells, while the PCSK9 inhibitor had no effect (Figures 4A, B). Conversely, under hypoxic conditions, PCSK9i-treated H5V cells proliferated faster relative to control (Figures 4A, B). Also, a slight increase in the proliferation rate was shown in H5V cells treated with atorvastatin under hypoxia (Figures 4A, B).

### 3.4 Effects of atorvastatin and PCSK9i on endothelial NO formation in H5V cells

**FIGURE 5:**
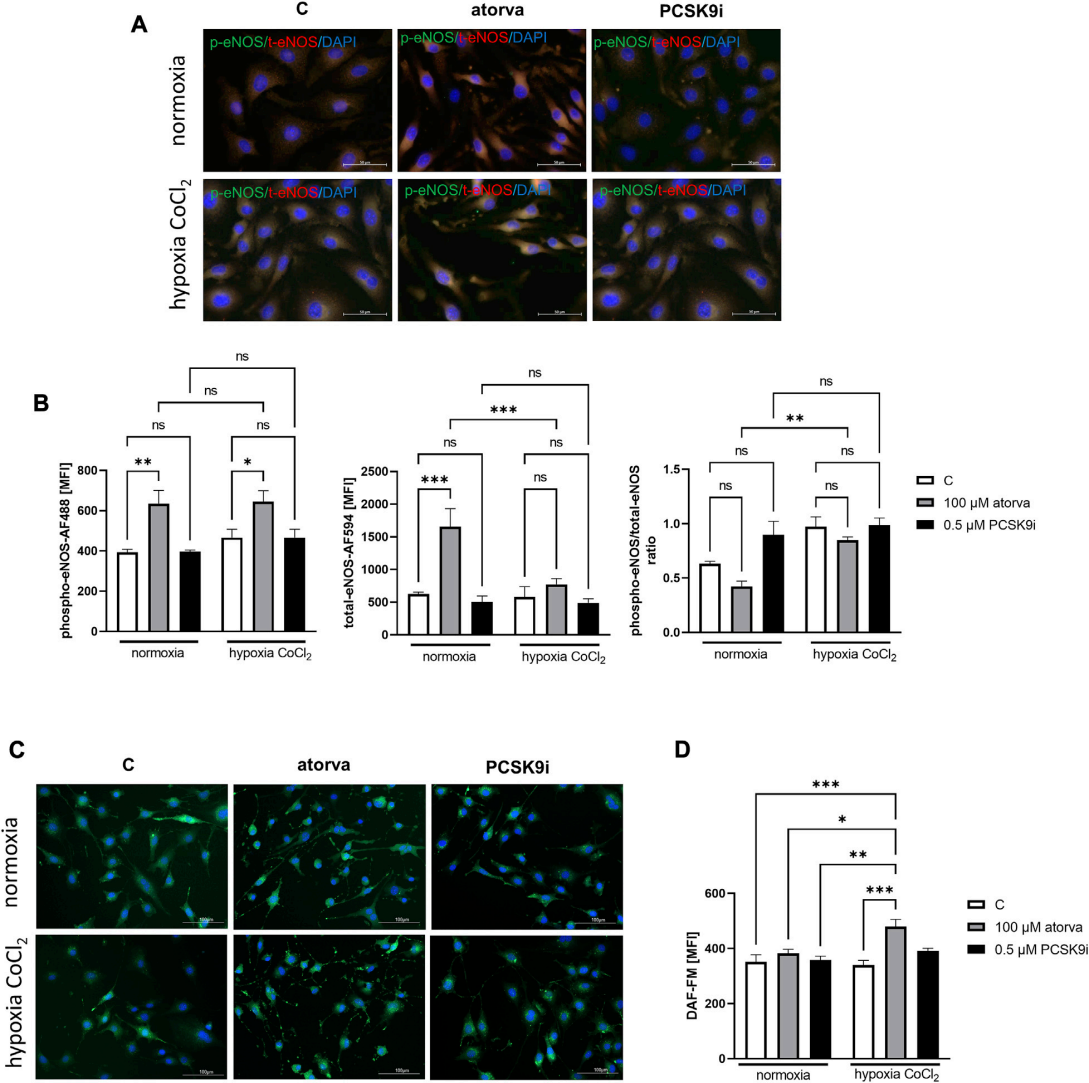
Nitric oxide production in murine heart endothelial cells (H5V) after treatment with atorvastatin and PCSK9i in normoxia and hypoxia-mimicking conditions. Representative images of the co-localization (yellow area) of phosphorylated endothelial nitric oxide synthase (phospho-eNOS) stained with Alexa Fluor 488 (green) and total eNOS stained with Alexa Fluor 594 (red) **(A)**. Quantitative analysis of mean fluorescence intensity (MFI) for phospho-eNOS, total-eNOS and phospho-eNOS/t-eNOS ratio in control (vehicle-pre-treated) cells and cells treated with 0.1 mM atorvastatin or 500 nM PCSK9 inhibitor in normoxia and CoCl_2_-mimicking hypoxia (24 h with 0.1 mM CoCl_2_) conditions **(B)**. Representative images of NO staining by DAF-FM (green) **(C)** and quantitative analysis of MFI for DAF-FM **(D)** in control (vehicle-pre-treated) cells and cells pre-treated with 100 µM atorvastatin or 0.5 µM PCSK9 inhibitor, after following incubation under normoxia and CoCl_2_-mimicking hypoxia (24 h with 0.1 mM CoCl_2_) **(B)** conditions. Values are shown as mean ± SEM; *n* = 6; *p < 0.05, **p < 0.01, ***p < 0.001.

Treatment of H5V cells with atorvastatin caused increased in phosphorylated endothelial nitric oxide synthase (phospho-eNOS) in comparison to control under both, normoxia and hypoxia-mimicking conditions (Figures 5A, B). Moreover, 24 h atorvastatin treatment H5V cells had stimulated total eNOS as compared to control cells (Figures 5A, B). Simultaneous treatment of H5V cells with 100 µM atorvastatin and 100 µM CoCl2 caused increase in fluorescence intensity of DAF-FM dye, used for detection of nitic oxide (Figure 5C, D). Treatment with PCSK9i did not affect phospho-eNOS, total-eNOS and NO levels in H5V cells compared to controls under both, normoxia and hypoxia mimicking conditions (Figures 5A–C).

### 3.5 Effect of atorvastatin and PCSK9i on intracellular nucleotide concentration in murine cardiomyocytes (HL-1 cells)

**FIGURE 6:**
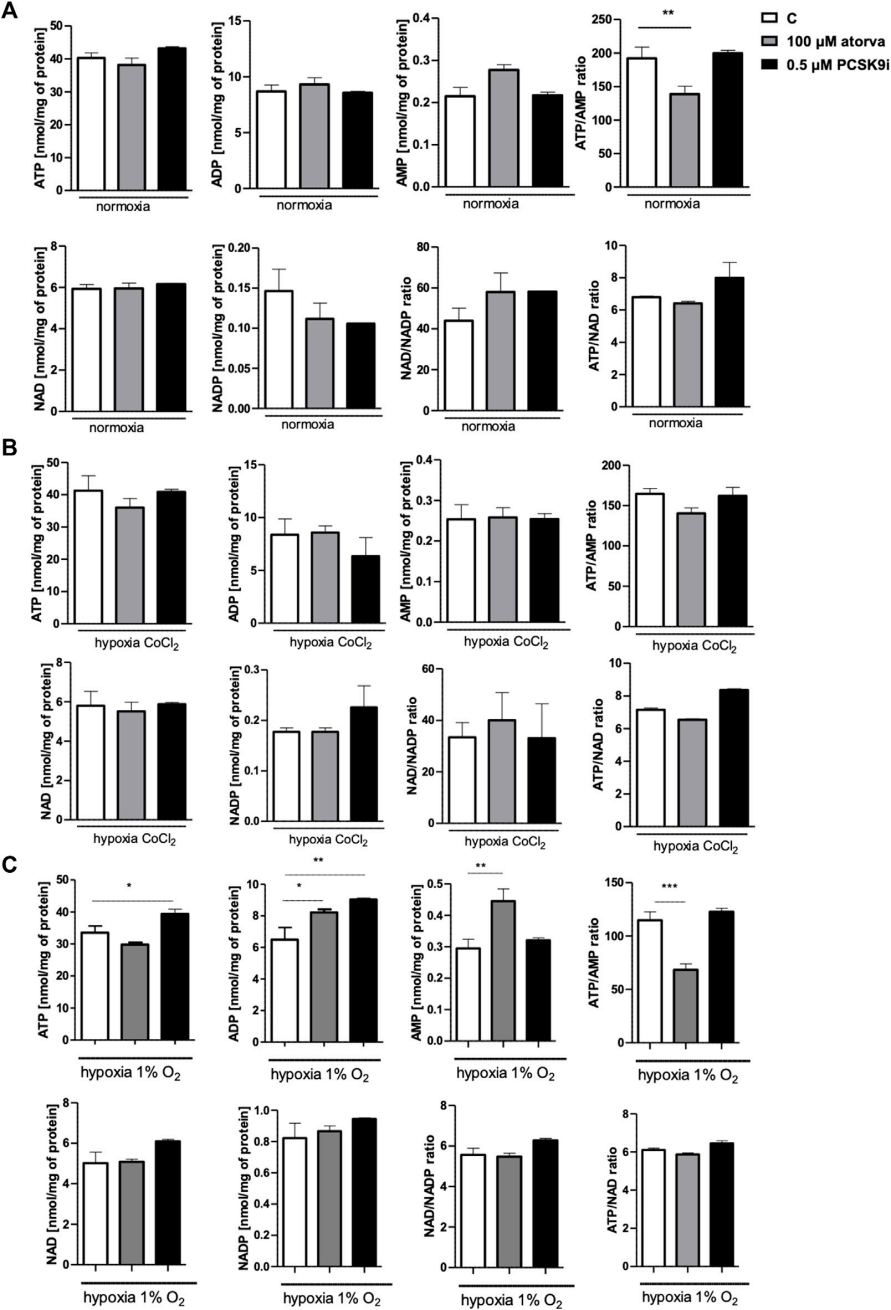
Intracellular nucleotide concentration in murine cardiomyocytes (HL-1) after atorvastatin and PCSK9 inhibitor (PCSK9i) treatment in normoxia, hypoxia-mimicking conditions (CoCl_2)_ and hypoxia (1% O_2_). The concentrations of adenosine triphosphate (ATP), adenosine diphosphate (ADP), adenosine monophosphate (AMP), nicotinamide adenine dinucleotide (NAD^+^), nicotinamide adenine dinucleotide phosphate (NADP) and the ratios of ATP/ADP and ATP/NAD^+^ after 100 µM atorvastatin and 0.5 µM PCSK9i treatment under normoxia **(A)**, CoCl_2_-mimicking hypoxia (0.1 mM CoCl_2_, 24 h) **(B)** and hypoxia (1% O_2_ in hypoxic chamber, 24 h) **(C)** conditions. Values are shown as mean ± SEM; *n* = 6; *p < 0.05, **p < 0.01, ***p < 0.001, ****p < 0.0001 vs. untreated cells in normoxia.

Determination of intracellular nucleotide concentration in murine cardiomyocytes after 100 µM atorvastatin treatment revealed decreased ATP/AMP ratio in comparison to control under normoxia conditions. The concentrations of adenine nucleotides as well as NAD and NADP remained unchanged after treatment with statin under both, normoxia and hypoxia-mimicking conditions (Figures 6A, B). Also, treatment with PCSK9 inhibitor has a minor effect on the concentration of nucleotides under these conditions (Figures 6A, B). Interestingly, after 24 h in 1% O2 hypoxia, ATP concentration was higher after PCSK9i treatment, while ATP/AMP ratio was lower after treatment with atorvastatin in comparison to normoxic conditions (Figure 6C).

### 3.6 Effect of atorvastatin and PCSK9i on mitochondria in HL-1 cells

**FIGURE 7:**
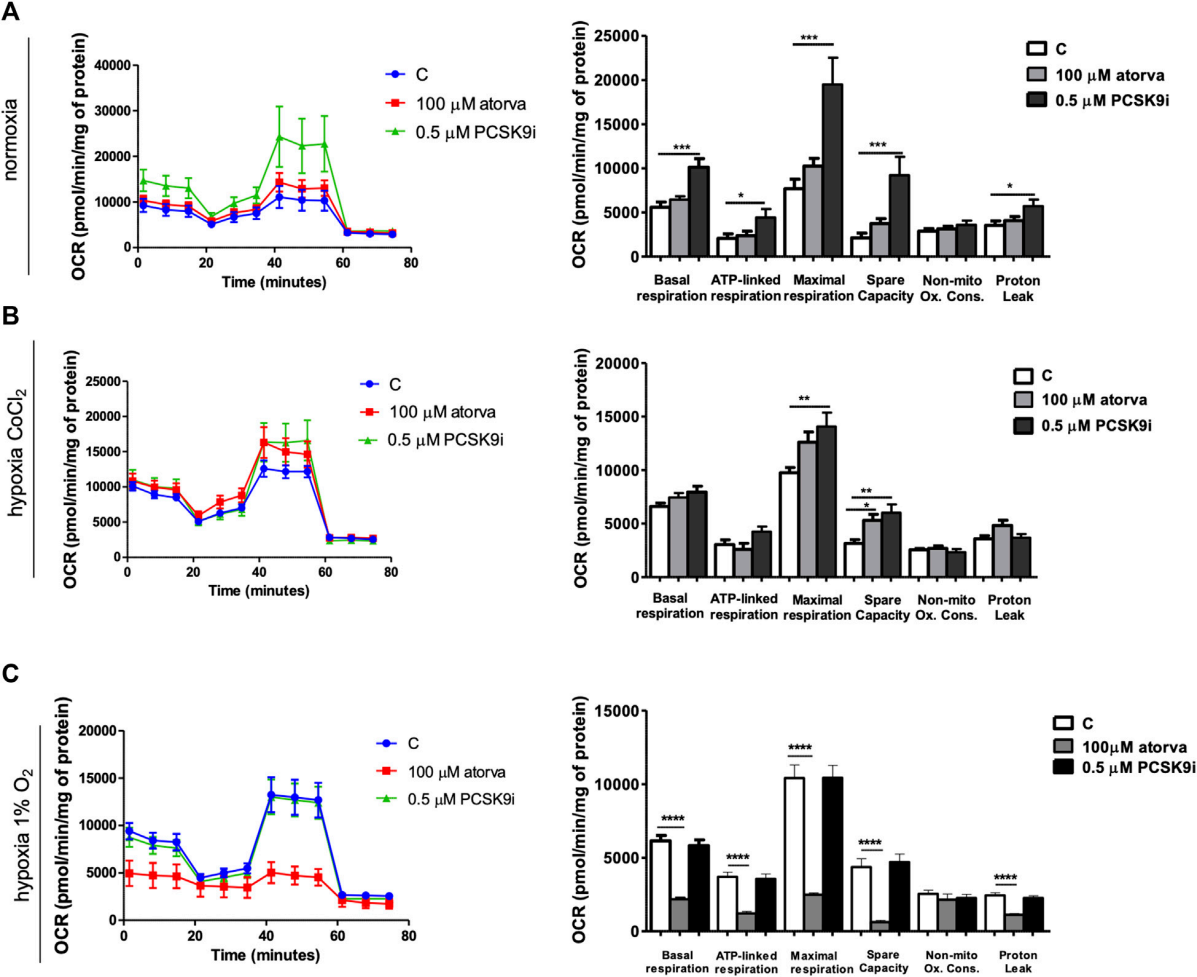
Mitochondria function in murine cardiomyocytes (HL-1) after atorvastatin and PCSK9 inhibitor (PCSK9i) treatment in normoxia, hypoxia-mimicking conditions and hypoxia (1% O_2_). Oxygen consumption rate (OCR) as presented by the Seahorse XFp Extracellular Flux Analyzer after the sequential injection of oligomycin, carbonyl cyanide-p-trifluoromethoxyphenylhydrazone (FCCP) and mix of rotenone and antimycin A, and parameters of mitochondrial function in control (vehicle-pre-treated) cells and cells pre-treated with 100 µM atorvastatin or 0.5 µM PCSK9 inhibitor, after following incubation in normoxia **(A)**, CoCl_2_-mimicking hypoxia (24 h with 0.1 mM CoCl_2_) **(B)** and hypoxia (1% O_2_ in hypoxic chamber, 24 h) **(C)** conditions. Values are shown as mean ± SEM; *n* = 4; *p < 0.05, **p < 0.01, ***p < 0.001.

Next, we examined the mitochondria function in murine cardiomyocytes (HL-1) treated with atorvastatin and PCSK9i using Seahorse analyzer. HL-1-treatment with PCSK9i improved mitochondrial function as compared to control under normoxia, while treatment with atorvastatin in the same conditions did not (Figure 7A). PCSK9i-treated HL-1 cells were characterized by improved basal respiration, ATP-linked respiration, maximal respiration, spare capacity and proton leak under normoxia (Figure 7B). However, under hypoxia-mimicked conditions, Hl-1 treatment with PCSK9i contributed to increased maximal respiration and spare capacity, while the treatment with atorvastatin stimulated only spare capacity as compared to control (Figure 7B). Ultimately, treatment of HL-1 cells with atorvastatin under hypoxic conditions (1% O2 in a hypoxic chamber) significantly downregulated mitochondria function, while PCSK9i maintained it (Figure 7C).

**FIGURE 8:**
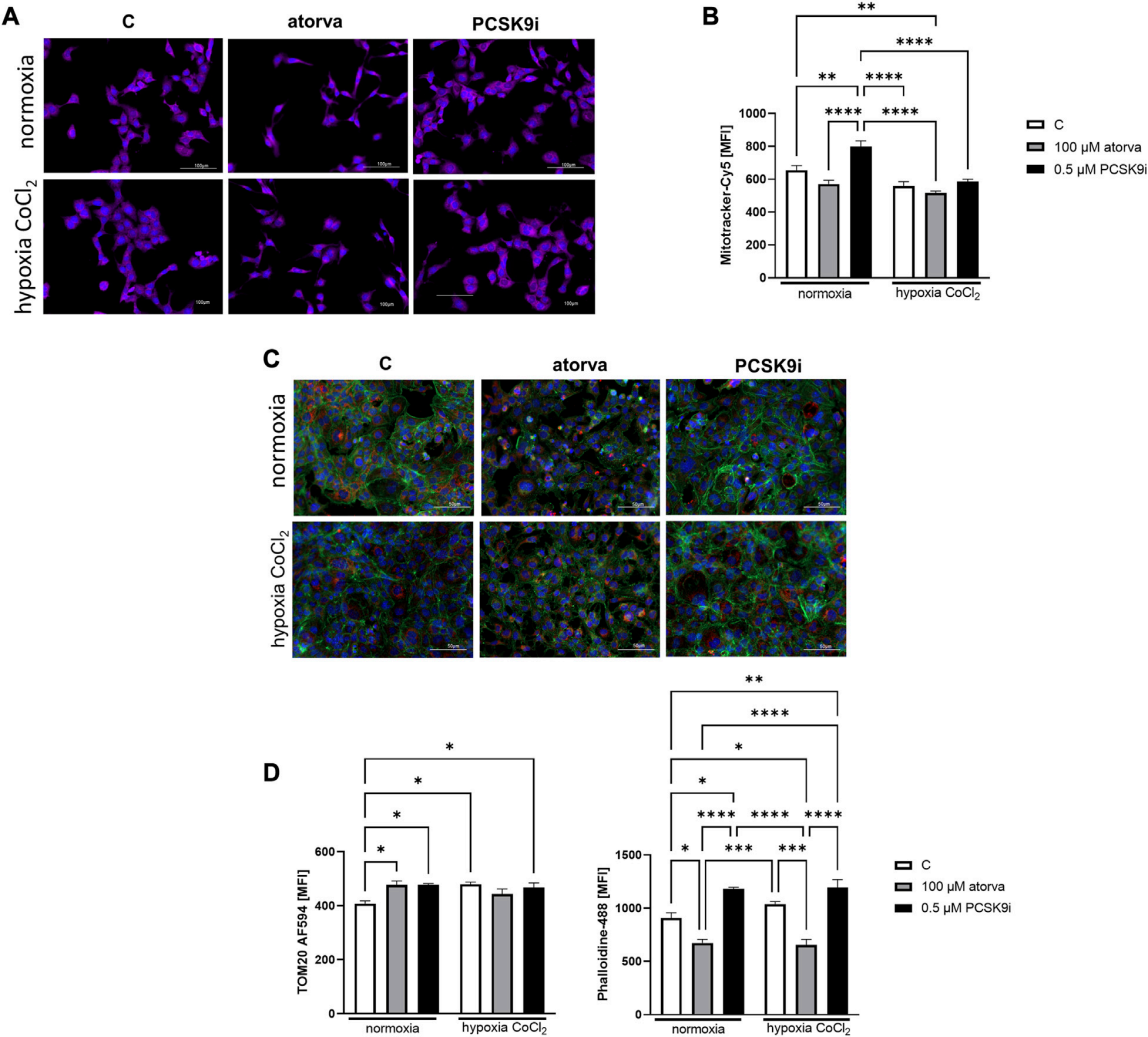
Mitochondria abundance and membrane potential in murine cardiomyocytes (HL-1) after atorvastatin and PCSK9 inhibitor (PCSK9i) treatment in normoxia and hypoxia-mimicking conditions. Representative images of Mitotracker-Cy5 fluorescence staining **(A)** and quantitative analysis of mean fluorescence intensity for Mitotracker-Cy5 in control (vehicle-pre-treated) cells and cells pre-treated with 100 µM atorvastatin or 0.5 µM PCSK9 inhibitor, after following incubation in normoxia and CoCl_2_-mimicking hypoxia (24 h with 0.1 mM CoCl_2_) **(B)** conditions. Representative images for TOM20-AF594 (red), α-actin-Phalloidin488 (green) and nuclei-DAPI (blue) staining analysed using fluorescence microscope **(C)** and quantitative analysis of MFI for TOM20-AF594 **(D)** and quantitative analysis of mean fluorescence intensity for α-actin-Phalloidin488 **(D)**. Values are shown as mean ± SEM; *n* = 6; **p < 0.01, ****p < 0.0001.

Furthermore, HL-1 cells treated with 0.5 µM PCSK9i, were characterized by increased mitochondrial membrane potential under normoxia as compared to control (Figures 8A, B), while atorvastatin treatment did not generate this improvement. Subsequently, we determined mitochondrial abundance by TOM20 protein immunofluorescence staining in HL-1 cells treated atorvastatin and PCSK9i under both conditions. Quantitative analysis of MFI revealed increased expression of TOM20 after cell treatment with PCSK9i as compared to atorvastatin-treated cells under normoxia and hypoxia-mimicking conditions, while treatment with atorvastatin did not change the mitochondria number (Figures 8C, D).

### 3.7 Effects of atorvastatin and PCSK9i treatment on α-actin cytoskeleton and proliferation rate in HL-1 cells

Analysis of actin filaments in the cytoskeleton by phalloidin staining of HL-1 cells was performed after treatment with atorvastatin and PCSK9 inhibitor under normoxia and hypoxia-mimicking conditions. Quantitative analysis of phalloidin MFI exposed the cytoskeleton disorganization in HL-1 cells treated with atorvastatin relative to control under both, normoxia and hypoxia-mimicking conditions (Figures 8C, E). Interestingly, after treatment with PCSK9i, we observed an improvement of cytoskeleton structure relative to control under both conditions (Figures 8C, E).

**FIGURE 9:**
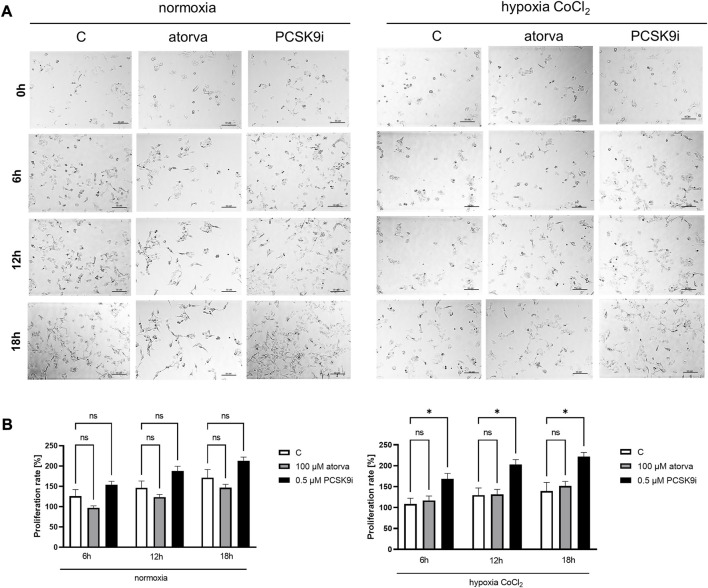
Proliferation rates of murine cardiomyocytes (HL-1) in the presence of atorvastatin and PCSK9 inhibitor (PCSK9i) in normoxia and hypoxia-mimicking conditions. Representative images **(A)** and quantitative results **(B)** of the proliferation rates of control (vehicle-pre-treated) cells and cells pre-treated with 100 µM atorvastatin or 0.5 µM PCSK9 inhibitor, after incubation in normoxia and CoCl_2_-mimicking hypoxia (24 h with 0.1 mM CoCl_2_) conditions. Values are shown as mean ± SEM; *n* = 6; *p < 0.05.

The proliferation test was conducted in HL-1 cells treated with PCSK9i or atorvastatin under normoxia and hypoxia-mimicking conditions using light microscope. Proliferation rate was unchanged in HL-1 cells after treatment with atorvastatin or PCSK9i in normoxia (Figures 9A, B). However, under hypoxic conditions, PCSK9i-treated HL-1 cell proliferated faster relative to control (Figures 9A, B), while atorvastatin treatment did not affect the proliferation of cardiomyocytes (Figures 9A, B).

## 4 Discussion

This study displayed a better mitochondrial respiration in mouse heart endothelial cells and cardiomyocytes after atorvastatin and PCSK9i treatment. Interestingly, these effects were observed after both pharmacological interventions only in hypoxia-mimicked conditions and were limited to the parameters related to the mitochondria response to acute cellular stress, which are the maximal respiration and spare capacity. PCSK9i were superior to statin under normoxic conditions improving basal and ATP-linked respiration in endothelial cells and cardiomyocytes. Moreover, as opposed to statin treatment, these parameters were also enhanced in hypoxic endothelial cells after PCSK9i. The improved respiratory parameters caused by PCSK9i were linked with better mitochondria abundance, its membrane potential, and intracellular adenine nucleotide status. In comparison to atorvastatin, PCSK9i did not cause intracellular drop of ATP, but maintained ATP/AMP ratio and total adenine nucleotide content, simultaneously improving proliferation rates of endothelial cells and cardiomyocytes. In addition, PCSK9i treatment did not destabilize the actin cytoskeleton as in the case of statins. However, PCSK9i failed to show some of the beneficial effects that were seen after statin treatment, such as an improvement in NO production by endothelium. We revealed increased eNOS phosphorylation after atorvastatin-treatment in H5V cells. One of the possible mechanism responsible for that observation is activation of the AMP-activated protein kinase (AMPK) pathway (Gorabi et al., 2019). It has been demonstrated that incubation of HUVECs with atorvastatin stimulates activity of AMPK and eNOS phosphorylation in dose and time-dependent manner (Sun et al., 2006). Interestingly, increased eNOS phosphorylation was also shown on bovine aortic endothelial cells treated with HMG-CoA reductase inhibitors, such as lovastatin and pravastatin (Harris et al., 2004).

Cardiac microcirculation is an essential component of the heart that regulates myocardial perfusion and thus meets the heart metabolic demand with energy substrates and oxygen delivery through the blood (Vancheri et al., 2020). Due to the cross talk with adjacent cardiomyocytes, cardiac microvascular endothelial cells control the signal transduction and energy supply by producing vasoactive, growth promoting, proangiogenic, and cytoprotective substances (Eelen et al., 2015; Lim et al., 2015). In addition to modulation of the vascular tone, a series of vasodilators, such as NO or bradykinin and vasoconstrictors, such as endothelin are released by endothelial cells to prevent platelet aggregation and blood clot formation (Van Hinsbergh, 2012). It has been shown that in the heart failure, in parallel to phenotypic changes in cardiomyocytes, such as cell hypertrophy or sarcomere reorganization, microvascular endothelial cells undergo a major shift from the key energy suppliers and effectors of angiogenesis into a matrix-secretory phenotype that eventually leads to fibrosis (Harvey and Leinwand, 2011; Trenson et al., 2021).

Mitochondria are crucial organelles for cell death by releasing pro-apoptotic factors and regulating the production of ATP (Guan et al., 2018). It was found that the mitochondria content in different types of heart cells varies due to their energy requirements. In this meaning, endothelial cells have relatively few mitochondria, approximately 2%–6% of cytoplasm volume, while cardiomyocytes more than 32% (Kluge et al., 2013). Unlike cardiomyocytes or skeletal muscle with higher energy demands, energy production by mitochondria is relatively low in quiescent endothelial cells, which primarily uses glycolysis to produce ATP (Davidson, 2010; Edwards et al., 2018). However in endothelial cells with angiogenic phenotype, such in case of cardiac endothelium, the contribution of oxidative phosphorylation in ATP production is higher as they need the fuel for the proliferation, migration, and maintaining of their structure (Du et al., 2021). However, in cardiac ischemia/reperfusion injury when the oxygen supply is impaired, mitochondrial respiration in coronary microcirculation decreases and glycolytic metabolism is enhanced to compensate for the reduction in ATP regeneration (Fitzgerald et al., 2018). Endothelial cells are prompted to glycolytic metabolism in hypoxia conditions by the HIF1α activation, that inhibits tricarboxylic acid-induced oxidative phosphorylation (Ullah and Wu, 2021). However, it has been demonstrated that glycolysis is not fully sufficient to regenerate cell ATP demands that triggers a series of consequences in endothelial cell function, including decreased production and release of endothelial-derived NO (Bierhansl et al., 2017). In this study, we used a HIF-1α stabilator to induce hypoxia-mimicking conditions in mouse cardiac endothelial cells and cardiomyocytes (Rinderknecht et al., 2021). Previously, we have shown that CoCl_2_ by the inhibition of the prolyl hydroxylase increases the HIF-1α concentration in mouse and human microvascular endothelial cells that was related to the decreased intracellular ATP concentration via diminished mitochondrial oxidative phosphorylation (Kutryb-Zajac et al., 2022a). In this work, we have shown that both atorvastatin and PCSK9i upregulated mitochondrial function in CoCl_2_-stimulated endothelial cells and cardiomyocytes. In particular, mitochondrial respiratory parameters related to the response to cellular stress were improved. Interestingly, the rates of basal and ATP-linked respiration were higher under both normoxia and hypoxia-mimicking conditions in endothelial cells and under hypoxia in cardiomyocytes only after PCSK9i treatment. Additionally, the highest tested concentration of PCSK9i (0.5 μM) counteracted the drop of intracellular ATP and ATP/AMP ratio induced by CoCl_2_. While, atorvastatin not only did not protect against the loss of ATP, but also caused its decrease in conditions of normoxia. These results underline the superior effect of PCSK9i over statins on the energy metabolism of cardiac endothelial cells and cardiomyocytes. Additionally, we revealed changes in mitochondrial morphology in H5V cells after treatment with PCSK9i and atorvastatin. It has been demonstrated that statins-treatment increase the size of isolated mitochondria from human T cells (Miettinen and Björklund, 2016) and induced a loss of outer mitochondrial membrane integrity in isolated mitochondria from human umbilical vein endothelial cells (EA.hy926 cell line) (Broniarek and Jarmuszkiewicz, 2018). Also, the altered mitochondrial morphology (with increased membrane density) were observed in cardiomyocytes treated with atorvastatin, which was directly linked with ferroptosis (Zhang et al., 2022). That was in line with our observations, where atorvastatin-treated H5V cells were characterized by swollen and disorganized mitochondria morphology. Moreover, we demonstrated elongated mitochondrial structure after treatment with PCSK9i in endothelial cells, which, according to current research, allows to maintain ATP production, increase dimerization and activity of mitochondrial Complex V (Gomes et al., 2011). As we have shown, the mechanism responsible for the better mitochondrial respiration after PCSK9i may be the improvement of mitochondrial membrane potential, as well as the effect on mitochondria biogenesis, since the content of outer membrane protein TOM20, a reliable marker of mitochondrial mass was increased after PCSK9 inhibition by synthetic LDL receptor EGF-A domain peptide (Contino et al., 2017). It has been shown that the EGF-A domain is critical for PCSK9 binding to LDL receptor at the cell surface and eventually leads to the receptor degradation (Gu et al., 2013). PCSK9 is an enzyme required for intra-cellular proteolytic cleavage and is mainly expressed by hepatocytes, but it was found that also other cells such as macrophages, endothelial cells, vascular smooth muscle cells (VSMC), or cardiomyocytes produce and release PCSK9 under stress conditions, like hypoxia (Yang et al., 2020; Wu et al., 2022). Thus, circulating PCSK9 protein prevents the uptake of LDL-c by the cells. This process can be reversed, by the inhibitors of PCSK9 binding to LDL receptor, such as synthetic peptides or monoclonal antibodies that increase LDL-c clearance by the cells and in consequence lowers LDL-c plasma concentration (Wu et al., 2022). Evolocumab, one of the FDA-approved PCSK9i is as a fully human monoclonal IgG2 antibody that revealed the beneficial effects on coronary endothelial function analysed by MRI that reflected NO-dependent endothelial vasodilatation in dyslipidemic patients (Leucker et al., 2020). Although in our study, we did not observe the direct effect of PCSK9i on endothelial cell NO production, the positive outcomes in evolocumab-treated patients may be the secondary effects of lipid-lowering therapy (Leucker et al., 2020). Interestingly, it has been found that PCSK9 inhibition by evolocumab displayed a cytoprotective effect directly on human endothelial cells *in vitro* and reduced ox-LDL-induced cardiomyocyte apoptosis *in vivo* (Safaeian et al., 2020; Li et al., 2022). Recently it has been revealed that evolocumab treatment counteracted the accumulation of mitochondrial ROS (mtROS) and inflammation in human aortic endothelial cells (TeloHAEC), and this phenomenon may be related to SIRT3 expression (D’Onofrio et al., 2023). In turn, the protective mechanisms of evolocumab treatment that were shown in cardiomyocytes, particularly emphasise the improvement of the mitochondrial function, which is in line with our results. It was demonstrated that evolocumab counteracted the harmful effects of ox-LDL, by decreasing of mtROS levels and mitochondrial fragmentation in neonatal mouse ventricular myocytes (Li et al., 2022). In other study, it was found that under oxLDL stimulation, HUVEC strongly upregulated PCSK9 protein expression, pyroptosis, and inflammatory factor release, and the silencing of PCSK9 expression suppressed the oxLDL-induced damage of HUVECs (Zeng et al., 2021). The authors have also shown that PCSK9 inhibition improved mitochondrial function and reversed mitochondrial membrane potential collapse via ubiquinol-cytochrome c reductase core protein 1 UQCRC1/ROS pathway.

In addition to monoclonal antibodies, a new promising drug for treating hypercholesterolemia via PCSK9 mechanism has been recently approved for clinical use. It is inclisiran, a long-acting synthetic small interfering ribonucleic acid, which targets PCSK9 mRNA (Lamb, 2021). Moreover, it is conjugated to N-acetylgalactosamine carbohydrates, that allows for precise uptake of the inclisiran by the cells, followed by suppression of gene expression. In comparison to monoclonal antibodies, it is assumed that siRNA PCSK9i may expand their pleiotropic effects well beyond interfering with PCSK9 binding to the LDL receptor on the cell surface. As cell membrane-penetrating agents, they affect intracellular PCSK9-dependent mechanisms that were shown as beneficial for cardiomyocytes via the modulation of NF-κB signaling, PI3K/AKT-SREBP-2 pathway, or ATM-LKB1-AMPK axis (Xu et al., 2023). Moreover, it has been found that VSMC cell-derived PCSK9 protein induces mtDNA damage and cell apoptosis via mtROS formation. While, PCSK9 inhibition by siRNA strongly reduced this damage in VSMC (Ding et al., 2016). Similar observations have been made in mouse models, where wild type mice showed dramatic PCSK9 release and mtDNA damage following LPS administration, in comparison to PCSK9 knock-out mice, which showed lesser mtDNA damage that correlated with slighter PCSK9 release (Ding et al., 2016). On the other side, it was demonstrated that PCSK9 reduces vascular endothelial cell uptake of LPS via LDLR-mediated endocytosis. Consequently, PCSK9 decreased the LPS-induced proinflammatory responses in vascular endothelial cells (Leung et al., 2022).

In our study, we noticed the faster proliferation rates and preservation of the actin cytoskeleton structure in PCSK9i-treated endothelial cells and also in cardiomyocytes. Except some data on the PCSK9 protein-dependent inhibition of vascular cell proliferation, little is known about the effects of PCSK9i on cell proliferation potential (Guo et al., 2022). Until recently, cardiomyocytes were considered as non-proliferating, with the exception of neonatal cells. However, according to current reports, adult cardiomyocyte proliferation occurs, and this phenomenon has a great potential for cardiac regeneration (Yester and Kühn, 2017). It has been also found that proliferation of endothelial cells can be crucial in both, cardiac and vascular regeneration and these mechanisms are related to the activity of mitochondrial respiratory chain complex III (Hong et al., 2016; Diebold et al., 2019). In addition, it should be emphasized that PCSK9i has the advantage over statins, which induce actin polymerization and compartmentalization (Sarkar and Chattopadhyay, 2022). PCSK9i maintained actin cytoskeleton undisturbed, which is essential for cell division and proliferation (Luo et al., 2020).

## 5 Conclusion

This study compared direct effects of atorvastatin and PCSK9i on the bioenergetics and function of mouse heart microvascular endothelial cell line (H5V) and mouse cardiomyocyte cell line (HL-1). We revealed an improvement in mitochondrial respiration and better intracellular adenine nucleotide content in cell stress conditions induced by CoCl_2_-mimicked hypoxia after PCSK9i treatment. It was related with better proliferation rates and maintained cytoskeleton structure of cardiac cells that may be of great importance for further experimental and clinical research on heart regeneration and vascular atherosclerosis.

## Data Availability

The original contributions presented in the study are included in the article, further inquiries can be directed to the corresponding author.
